# Supplementary material for: miR-20a is upregulated in serum from domestic feline with PKD1 mutation
Source: PLoS One. 2022 Dec 20;17(12):e0279337. doi: 10.1371/journal.pone.0279337 (PMC9767353; doi:10.1371/journal.pone.0279337)
Supplement: S1 File — (PDF) [file pone.0279337.s001.pdf]

Nº:_______

(ENGLISH)

Research Informed Consent Form

I,____ ID Number:_____________________________.
Born City:____________________ Date of Birth: ___/___/______ Marital Status:____________________.
Occupation:______________________ Address:____.
Owner of (cat's name): ____.

I am being invited to participate in a study called **The use of microRNA for the diagnosis of diseases in domestic dogs and cats**, whose objectives and justifications are to investigate the expression of microRNAs in samples of domestic dogs and cats, that can be correlated with a clinical profile for important diseases in veterinary medicine. Besides that, this study has a potential to identify new biomarkers that can be use for early molecular diagnosis techniques. After analyzing the results, the characterization of the trials will allow innovation for biotechnological resources, related to the development of efficient and less invasive techniques that can be used in the clinical routine.

My part in the study will be to allow the veterinarian team to collect 3 to 5 mL of peripheral blood, and 10 mL of urine from my cat, for further analysis.

I declare that I have received the information about any discomfort that the cat may feel during the blood collect procedure. Any study results will be available after tests and trials are complete.

I am aware that my privacy will be respected, my name or any other data or element, that may in any way identify me, will be kept confidential.

I have also been informed that I may refuse to participate in the study, or withdraw my consent at any time, without having to justify. Even if I wish to leave the research, I will not suffer any harm to the assistance I have been receiving.

This project is under the responsibility of Professor Giane Regina Paludo (PhD. coordinator) and will involve other DSc. and students of this institution.

With knowledge and understanding about the content, and purpose, of this study, I express my free consent to participate, being fully aware that there is no economic value, to be received or to be paid, for my participation.

Brasilia, Date: ______________________

____

Signature

Contact:

Professor Giane Regina Paludo (PhD. DSc)
Coordinator
College of Agronomy and Veterinary Medicine
University of Brasilia, ICC Centro, Campus Universitário Darcy Ribeiro, Brasilia, DF, Brazil – CP 04508 – CEP 70910-900
e-mail: giane@unb.br
Phone number: (61) 3107-2850 / (61) 99556-7740

Nº:_______

Feline data:

Identification (number)/Name:

Race:

Age:

Sex:

Clinical Signs:

Is the animal under any treatment?:

Which?:

Clinical Suspicion:

Ultrasound (US) Report:

Nº:_______

(PORTUGUESE)

Termo de Consentimento Livre e Esclarecido

Eu,____RG:__________________.
Natural de:___________________nascido(a) em: ___/___/_____ estado civil:_________________,
Profissão:_____________________Endereço:____,
Proprietário(a) do animal (nome do animal): ____

Estou sendo convidado a participar de um estudo denominado **Uso do microRNA para diagnóstico de doenças de cães e gatos domésticos**, cujos objetivos e justificativas são investigar a expressão de microRNAs em amostras de cães e gatos domésticos correlacionadas ao perfil clínico de doenças com importância na medicina veterinária, com possível identificação de novos biomarcadores para diagnóstico precoce através de técnicas moleculares (Reação em Cadeia Polimerase em Tempo Real). Após a análise dos resultados, a caracterização dos ensaios possibilitará a inovação dos recursos biotecnológicos aliados ao desenvolvimento de técnicas eficientes e menos invasivas que possam ser usadas na rotina clínica.

Minha participação no estudo será permitir que a equipe realizadora do projeto colha de 3 a 5 mL de sangue, para posterior análise.

Declaro ter recebido os esclarecimentos necessários sobre possíveis desconfortos decorrentes do estudo, levando-se em conta que é uma pesquisa, e os resultados positivos ou negativos somente serão obtidos após sua realização. Assim, o animal pode apresentar um desconforto temporário no local da colheita de sangue, podendo ainda apresentar desconforto e irritação durante a colheita.

Estou ciente de que minha privacidade será respeitada, ou seja, meu nome ou qualquer outro dado ou elemento possa, de qualquer forma, me identificar, será mantido em sigilo.

Também fui informado de que posso me recusar a participar do estudo, ou retirar meu consentimento a qualquer momento, sem precisar justificar, e de, por desejar sair da pesquisa, não sofrerei qualquer prejuízo à assistência que venho recebendo.

O referido projeto será conduzido sob responsabilidade da Profª. Drª. Giane Regina Paludo (coordenadora) e envolverá outros docentes e alunos desta instituição.

Tendo sido orientado quanto ao teor de todo o aqui mencionado e compreendido a natureza e o objetivo do já referido estudo, manifesto meu livre consentimento em participar, estando totalmente ciente de que não há nenhum valor econômico, a receber ou a pagar, por minha participação.

Brasília, de de 20 .

____
Assinatura Proprietário

Contato:

Professora Dra. Giane Regina Paludo
Coordenadora do Projeto
Faculdade de Agronomia e Medicina Veterinária
ICC Centro – CP 04508 – CEP 70910-900
e-mail: giane@unb.br
Telefones para contato: (61) 3107-2850 / 99556-7740

____

Nº:_______

Dados do felino:

Identificação (número)/Nome:

Raça:

Idade:

Sexo:

Sinais Clínicos:

Animal está sob algum tratamento?:

Qual?:

Suspeita Clínica:

Laudo de US:
