# Supplementary material for: miR-20a is upregulated in serum from domestic feline with PKD1 mutation
Source: PLoS One. 2022 Dec 20;17(12):e0279337. doi: 10.1371/journal.pone.0279337 (PMC9767353; doi:10.1371/journal.pone.0279337)
Supplement: S1 Protocol — (PDF) [file pone.0279337.s002.pdf]

protocols.io

NOV 05, 2022

SHARE

WORKS FOR ME 1

Extraction-Protocol-miRNA-SCALONMC

DOI
dx.doi.org/10.17504/protocols.io.ewov1oozolr2/v1

Marcela Scalon[1], Giane Regina Paludo[1], Christine Souza Martins[1], Ricardo Titze de Almeida[1], Gabriel Ginani Ferreira[1], Franciele Schlemmer[1]

[1]Universidade de Brasília

Marcela Scalon

Marcela Scalon
Universidade de Brasília

COMMENTS 0

ABSTRACT

This protocol is intended as a guideline for the purification of total miRNAs, from serum and plasma, using the miRNeasy Mini Kit (QIAGEN cat. no. 217004). This is an adaptation of QIAGEN Supplementary Protocol for total RNA. The plasma samples were obtained from peripheral blood collection, stored in K3EDTA-containing tubes. The serum samples were obtained from peripheral blood collection, stored in non-additives tubes. Moreover, a synthetic spike-in control was included as a normalizing control of all purifications. The aim of this protocol is to provide purified miRNA material with quality and efficiency for qPCR procedures.

DOI

dx.doi.org/10.17504/protocols.io.ewov1oozolr2/v1

PROTOCOL CITATION

Marcela Scalon, Giane Regina Paludo, Christine Souza Martins, Ricardo Titze de Almeida, Gabriel Ginani Ferreira, Franciele Schlemmer 2022. Extraction-Protocol-miRNA-SCALONMC. **protocols.io**
https://dx.doi.org/10.17504/protocols.io.ewov1oozolr2/v1

KEYWORDS

miRNA Purification, Plasma miRNA, Serum miRNA, Spike-in synthetic control

LICENSE

This is an open access protocol distributed under the terms of the Creative Commons Attribution License, which permits unrestricted use, distribution, and reproduction in any medium, provided the original author and source are credited

CREATED

Nov 03, 2022

LAST MODIFIED

Nov 05, 2022

PROTOCOL INTEGER ID

72245

MATERIALS TEXT

- miRNeasy Mini Kit (QIAGEN cat. no. 217004)
- QIAzol Lysis Reagent

Equipment and reagents to be supplied in addition to the kit:

- Chloroform (without added isoamyl alcohol)
- Ethanol (70% and 96–100%); do not use denatured alcohol, which contains other substances such as methanol and methylethylketone
- Sterile, RNase-free pipet tips
- 1.5 ml or 2 ml microcentrifuge tubes
- Microcentrifuge(s) (with rotor for 2 ml tubes) for centrifugation at 4°C and at room temperature (15–25°C)
- Disposable gloves
- miScript Serum/Plasma Spike-In Control (cel-miR-39p) for normalization and/or as an internal control

SAFETY WARNINGS

When working with chemicals, always wear a suitable lab coat, disposable gloves, and protective goggles. For more information, consult the appropriate material safety data sheets (MSDSs), available from the product supplier.

BEFORE STARTING

- After collection and centrifugation, plasma or serum can be stored at 2–8°C for up to 6 hours or used directly in the procedure. For long-term storage, freezing at –20°C or –80°C in aliquots is recommended. Avoid prolonged incubation, which may compromise RNA integrity.

- Except for phase separation (step 7), all protocol and centrifugation steps should be performed at room temperature.

- The procedure is suitable for use with serum samples or with plasma samples containing either citrate or EDTA. Plasma samples containing heparin should not be used because this anticoagulant can interfere with downstream assays, such as RTPCR.

- Buffers RWT and RPE are supplied as concentrates. Before using for the first time, add the required volumes of ethanol (96–100%), as indicated on the bottle, to obtain a working solution.

- The Syn-cel-miR-39 miScript miRNA Mimic (cat. no. MSY0000010) can be added to samples to control for variations during the preparation of total RNA and subsequent steps, as it shows no sequence homology to any known human, mouse, or rat miRNA. miScript miRNA Mimics are provided lyophilized. Prepare the miScript miRNA Mimic stock and working solution as follows:

Stock - Add 300µl RNase-free water to lyophilized miScript Serum/Plasma Spike-In Control (10pmol). Final concentration will be 2 x 10^{10} copies/µl.

Dilution - Add 4µl stock (2 x 10^{10} copies/µl) to 16µl RNase-free water. Final concentration will be 4 x 10^{9} copies/µl.

Working solution for RNA purification - Add 2µl of 4 x 10^{9} copies/µl dilution to 48µl RNase-free water. Final concentration will be 1.6 x 10^{8} copies/µl.

1 Prepare serum or plasma or thaw frozen samples:
- Aliquots has to have 100µl of plasma or serum.

2 Add 500µl of QIAzol Lysis Reagent. Mix by vortexing or pipetting up and down.
Note: After addition of QIAzol Lysis Reagent, lysates can be stored at -70ºC for severa months.

3 Place the tube containing the homogenate on the benchtop at room temperature (15-25ºC) for 5 minutes.
Note: This step promotes dissociation of nucleoprotein complexes.

4 Optional: Add 3.5µl of the working solution of Syn-cel-miR-39 miScript miRNA Mimic.

5 Add 1 volume chloroform to the tube containing the homogenate and close securely. Vortex the tube vigorously for 15 seconds.
Note: Thorough mixing is important for subsequent phase separation.

6 Place the tube containing the homogenate on the benchtop at room temperature for 2-3 minutes.

7 Centrifuge for 15 minutes at 12,000 x g at 4°C. After centrifugation, heat the centrifuge up to room temperature (15-25°C) if the same centrifuge will be used for the next centrifugation steps.

After centrifugation, the sample separates into 3 phases: an upper, colorless, aqueous phase containing RNA; a white interphase; and a lower, red, organic phase.

8 Transfer the upper aqueous phase to a new collection tube (supplied in the kit).

Avoid transfer of any interphase material. Add 1.5 volumes of 100% ethanol and mix thoroughly by pipetting up and down several times. Do not centrifuge. Continue without delay with step 9.

Write down the volume of the aqueous phase, multiply it by 1.5 to find the volume of ethanol to be added.

A precipitate may form after addition of ethanol, but this will not affect the procedure.

9 Pipet up to 700 µl of the sample, including any precipitate that may have formed, into an RNeasy Mini spin column in a 2 ml collection tube (both supplied in kit). Close the lid gently and centrifuge at ≥8000 x g (≥10,000 rpm) for 15 s at room temperature (15–25°C). Discard the flow-through.*

Reuse the collection tube in step 10.

* Flow-through contains QIAzol Lysis Reagent and is therefore not compatible with bleach.

10 Repeat step 9 using the remainder of the sample. Discard the flow-through.*

Reuse the collection tube in step 11.

* Flow-through contains QIAzol Lysis Reagent and is therefore not compatible with bleach.

11 Add 700 µl Buffer RWT to the RNeasy Mini spin column. Close the lid gently and centrifuge for 15 s at ≥8000 x g (≥10,000 rpm) to wash the column. Discard the flowthrough.*

Reuse the collection tube in step 12.

* Flow-through contains Buffer RWT and is therefore not compatible with bleach.

12 Pipet 500 µl Buffer RPE onto the RNeasy Mini spin column. Close the lid gently and centrifuge for 15 s at ≥8000 x g (≥10,000 rpm) to wash the column. Discard the flow-through.

Reuse the collection tube in step 13.

13 Repeat step 12.

Note: Following centrifugation, remove the RNeasy Mini spin column from the collection tube carefully so the column does not contact the flow-through. Otherwise, carryover of ethanol will occur.

14 Place the RNeasy Mini spin column into a new 2 ml collection tube (not supplied in kit), and discard the old collection tube with the flow-through. Centrifuge in a microcentrifuge at full speed for 2 min.

The long centrifugation step dries the spin column membrane, ensuring that no ethanol is carried over during RNA elution.

Residual ethanol may interfere with downstream reactions.

15 Transfer the RNeasy Mini spin column to a new 1.5 ml collection tube (supplied in kit). Pipet 30–50 µl RNase-free water directly onto the RNeasy Mini spin column membrane. Close the lid gently and centrifuge for 1 min at ≥8000 x g (≥10,000 rpm) to elute the RNA.

Note: for a better miRNA recovery and higher miRNA concentration, use the eluted miRNA solution to make a second centrifuge step, in the same spin column, for 1 min at ≥8000 x g (≥10,000 rpm).
