# Supplementary material for: miR-20a is upregulated in serum from domestic feline with PKD1 mutation
Source: PLoS One. 2022 Dec 20;17(12):e0279337. doi: 10.1371/journal.pone.0279337 (PMC9767353; doi:10.1371/journal.pone.0279337)
Supplement: S2 Protocol — (PDF) [file pone.0279337.s003.pdf]

protocols.io

NOV 07, 2022

SHARE

WORKS FOR ME 1

Quantification-Protocol-miRNA-SCALONMC

DOI
dx.doi.org/10.17504/protocols.io.x54v9dd3pg3e/v1

Marcela Scalon[1], Christine Souza Martins[1], Gabriel Ginani Ferreira[1], Franciele Schlemmer[1], Ricardo Titze de Almeida[1], Giane Regina Paludo[1]

[1]Universidade de Brasília

Marcela Scalon
Universidade de Brasília

COMMENTS 0

ABSTRACT

This protocol is intended as a guideline for the quantification of total miRNAs, from serum and plasma samples, using the Qubit® microRNA Assay Kit and the Qubit® 2.0 Fluorometer. This method have been able to reproducibly quantify small RNA in pure samples at levels as low as 0.5 ng in the assay tube following. The assay detects all types of small RNA, including microRNA and siRNA, both single stranded and double stranded. Samples for quantification are the final product of purification procedures, stored in 1,5ml microtubes at -80ºC. The aim of this protocol is to provide the exact amount of each sample to standardize the dilution for RT-rtPCR protocol.

DOI

dx.doi.org/10.17504/protocols.io.x54v9dd3pg3e/v1

PROTOCOL CITATION

Marcela Scalon, Christine Souza Martins, Gabriel Ginani Ferreira, Franciele Schlemmer, Ricardo Titze de Almeida, Giane Regina Paludo 2022. Quantification-Protocol-miRNA-SCALONMC. **protocols.io**
https://dx.doi.org/10.17504/protocols.io.x54v9dd3pg3e/v1

LICENSE

This is an open access protocol distributed under the terms of the Creative Commons Attribution License, which permits unrestricted use, distribution, and reproduction in any medium, provided the original author and source are credited

CREATED

Nov 03, 2022

LAST MODIFIED

Nov 07, 2022

PROTOCOL INTEGER ID

72270

GUIDELINES

The assay accurately detects as little as 0.5 ng of small RNAs and has a dynamic range of 5 ng/mL to 500 ng/mL (1–100 ng) in the core assay range. The assay is accurate for initial sample concentrations from 0.05 ng/µL to 100 ng/µL. The kit provides concentrated assay reagent, dilution buffer, and pre-diluted small RNA standards.

MATERIALS TEXT

- Qubit® microRNA Reagent
- Qubit® microRNA Buffer
- Qubit® microRNA Standard #1
- Qubit® microRNA Standard #2
- Plastic container (disposable) for mixing the Qubit® working solution (step 2.3)
- Qubit® 2.0 Fluorometer
- Qubit® assay tubes 0,5ml (500 tubes, Cat. no. Q32856) or Axygen® PCR-05-C tubes 0,5ml (VWR, part no. 10011-830)
- Plasma and Serum Samples (2µl each)
- 1,5mL microtubes;
- Micropipettes and tips 10µl, 200µl, and 1000µl.

SAFETY WARNINGS

When working with chemicals, always wear a suitable lab coat, disposable gloves, and protective goggles. For more information, consult the appropriate material safety data sheets (MSDSs), available from the product supplier.

BEFORE STARTING

- If the reagents and samples are stored at a temperature colder than room temperature, it is important to warm it to room temperature and vortex briefly prior to use.

- Make sure the Qubit® 2.0 Fluorometer has the updated microRNA assay.qbt file uploaded.

- Use appropriate RNAse-free handling techniques, including RNAse-free gloves, pipette tips, and tubes. Keep the tube lids closed whenever possible; do not touch the pipet to the inside wall of the tube when withdrawing a sample,

1 Prepare the Qubit® working solution by diluting the Qubit® microRNA reagent 1:200 in Qubit® microRNA buffer as follows:

- 1µl of Qubit® microRNA reagent for each sample/standard
- 199µl of Qubit® microRNA buffer for each sample/standard

Note: Use a clean plastic tube each time you make Qubit® working solution. Do not mix the working solution in a glass container.

2 Prepare tube for Standard #1
Label the lid

Add:

- 190µl of the Working Solution prepared at STEP 1
- 10µl of Qubit® microRNA Standard #1

Mix by vortexing 2–3 seconds, being careful not to create bubbles.

Notes:
- Careful pipetting is critical to ensure that exactly 10 µL of Qubit® microRNA standard #1 is added to 190 µL of Qubit® working solution.
- Use only thin-wall, clear 0.5-mL optical-grade real-time PCR tubes. Acceptable tubes include Qubit® assay tubes (500 tubes, Cat. no. Q32856) or Axygen® PCR-05-C tubes (VWR, part number 10011-830).

3 Prepare tube for Standard #2
Label the lid

Add:

- 190µl of the Working Solution prepared at STEP 1
- 10µl of Qubit® microRNA Standard #2

Mix by vortexing 2–3 seconds, being careful not to create bubbles.

Notes:
- Careful pipetting is critical to ensure that exactly 10 µL of Qubit® microRNA standard #2 is added to 190 µL of Qubit® working solution.
- Use only thin-wall, clear 0.5-mL optical-grade real-time PCR tubes. Acceptable tubes include Qubit® assay tubes (500 tubes, Cat. no. Q32856) or Axygen® PCR-05-C tubes (VWR, part number 10011-830).

4 Prepare the tubes for Samples
Label the lid of each sample as needed

For each sample in each tube, add as follows:

- 198µl of the Working Solution prepared at STEP 1
- 2µl of sample (purified miRNA)

Mix by vortexing 2–3 seconds, being careful not to create bubbles.

The final volume in each tube should be 200μl

Note: Use only thin-wall, clear 0.5-mL optical-grade real-time PCR tubes. Acceptable tubes include Qubit® assay tubes (500 tubes, Cat. no. Q32856) or Axygen® PCR-05-C tubes (VWR, part number 10011-830).

5 Allow all tubes to incubate at room temperature for 2 minutes.

6 Proceed to “Read standards and samples”:

6.1 On the Home Screen of the Qubit® 2.0 Fluorometer, press microRNA.

The “Standards Screen” will be automatically displayed.

6.2 On the Standards screen, press Yes to read the standards.

6.3 Insert the tube containing Standard #1 into the sample chamber, close the lid, then press Read.

When the reading is complete (~3 seconds), remove Standard #1.

6.4 Insert the tube containing Standard #2 into the sample chamber, close the lid, then press Read.

When the reading is complete, remove Standard #2.

When the calibration is complete, the instrument displays the Sample screen.

6.5 Insert a sample tube into the sample chamber, close the lid, then press Read.

When the reading is complete (~3 seconds), remove the sample tube.

Note: The instrument displays the results on the Sample screen in ng/mL.
The value displayed corresponds to the concentration after your sample was diluted into the assay tube.

To find the concentration of the samples as ng/µl, use the “Dilution Calculator” feature of the Qubit® 2.0 Fluorometer as follows:

- Press Calculate Stock Conc. The “Dilution Calculator” screen containing the volume roller wheel is displayed.

- Using the volume roller wheel, select the volume of your original sample as 2µl. When you stop scrolling, the Qubit® 2.0 Fluorometer calculates the original sample concentration based on the measured assay concentration.

- To change the units in which the original sample concentration is displayed, press ng/mL. A pop-up window showing the current unit selection (as indicated by an adjacent red dash) opens. Select the unit ng/µl. To close the unit selection pop-up window, touch anywhere on the screen outside the pop-up.

- To exit the Dilution Calculator screen, press any navigator button on the bottom of the screen or Read Next Sample.

6.6 Repeat step 6.5 until all samples have been read.
