# Supplementary material for: miR-20a is upregulated in serum from domestic feline with PKD1 mutation
Source: PLoS One. 2022 Dec 20;17(12):e0279337. doi: 10.1371/journal.pone.0279337 (PMC9767353; doi:10.1371/journal.pone.0279337)
Supplement: S3 Protocol — (PDF) [file pone.0279337.s004.pdf]

protocols.io

NOV 08, 2022

SHARE

WORKS FOR ME 1

ReverseTranscription-Protocol-miRNA-SCALONMC

DOI
dx.doi.org/10.17504/protocols.io.261ge33j7l47/v1

Marcela Scalon[1], Christine Souza Martins[1], Gabriel Ginani Ferreira[1], Franciele Schlemmer[1], Ricardo Titze de Almeida[1], Giane Regina Paludo[1]

[1]Universidade de Brasília

Marcela Scalon

Marcela Scalon
Universidade de Brasília

COMMENTS 0

ABSTRACT

This protocol is intended as a guideline to perform the Reverse Transcription procedure to detect and quantify miRNAs. This method use TaqMan® MicroRNA Assays and Applied Biosystems real-time PCR instruments. The assays can detect and quantify small RNA in 1 to 10 ng of total RNA with a dynamic range of greater than six logs. This protocol works on detecting microRNA from samples that were purified from plasma and serum. All samples were stored at -80ºC until the realization of the RT procedure. The aim of this protocol is to use a miRNA-specifc, stem-loop RT primer, in order to produce cDNA from miRNA samples. The amplified products of RT procedure will then be used to perform qPCR or rtPCR.

DOI

dx.doi.org/10.17504/protocols.io.261ge33j7l47/v1

PROTOCOL CITATION

Marcela Scalon, Christine Souza Martins, Gabriel Ginani Ferreira, Franciele Schlemmer, Ricardo Titze de Almeida, Giane Regina Paludo 2022. ReverseTranscription-Protocol-miRNA-SCALONMC. **protocols.io** https://dx.doi.org/10.17504/protocols.io.261ge33j7l47/v1

LICENSE

This is an open access protocol distributed under the terms of the Creative Commons Attribution License, which permits unrestricted use, distribution, and reproduction in any medium, provided the original author and source are credited

CREATED

Nov 07, 2022

LAST MODIFIED

Nov 08, 2022

PROTOCOL INTEGER ID

72354

GUIDELINES

In the reverse transcription (RT) step, cDNA is reverse transcribed from total RNA samples using a small RNA-specific, stem-loop RT primer from the TaqMan® Small RNA Assays and reagents from the TaqMan® MicroRNA Reverse Transcription Kit.

Prepare the qPCR reactions in an area free of artificial templates.

When preparing samples for PCR amplification:

- Use a positive-displacement pipette or aerosol-resistant pipette tips.
- Follow proper pipette-dispensing techniques to prevent aerosols.
- Wear clean gloves and a clean lab coat (not previously worn while handling amplified PCR products or used during sample preparation).
- Change gloves whenever you suspect that they are contaminated.
- Maintain separate areas and dedicated equipment and supplies for:
    - Sample preparation
    - PCR setup
    - PCR amplification
    - Analysis of PCR products
- Never bring amplified PCR products into the PCR setup area.
- Open and close all sample tubes carefully. Centrifuge tubes before opening. Try not to splash or spray PCR samples.
- Keep reactions and components capped as much as possible.
- Clean lab benches and equipment periodically with 10% bleach solution.

MATERIALS TEXT

Kits and Reagents:

- TaqMan® MicroRNA Reverse Transcription Kit
- TaqMan® MicroRNA Assays:
    - Tube containing small RNA-specific RT primer

Required laboratory materials and equipment:

- Centrifuge with plate holders
- Disposable gloves
- Microcentrifuge
- Pipettors (positive-displacement, air-displacement, or multi-channel) and tips:
    - 1- to 20-µL range, 20- to 200-µL range, 100- to 1000-µL range
- Polypropylene tubes
- RNase-free, sterile-filtered water
- Vortexer

SAFETY WARNINGS

Minimize contact with chemicals. Wear appropriate personal protective equipment when handling chemicals (for example, safety glasses, gloves, or protective clothing).
Minimize the inhalation of chemicals. Do not leave chemical containers open. Use only with adequate ventilation (for example, fume hood).
Comply with all local, state/provincial, or national laws and regulations related to chemical storage, handling, and disposal.

BEFORE STARTING

Just before RT, and right after thawing of the samples, the step of quantification must be performed. Within the concentration values of each sample in hand, it is necessary to dilute all samples to the lowest value in order to level all concentrations that will run in the same set.

Allow the kit components to thaw on ice.

Prepare de RT mix

1 Allow the reagents and samples to thaw on ice.

Before use, mix gently and centrifuge briefly.

2 In a polypropylene 0,6 tube, prepare the RT mix on ice by scaling the volumes listed below to the desired number of RT reactions + 1 (excess volume to compensate for losses that occur during pipetting):

A	B
Component	Mix volume per 15-µL reaction*
Nuclease-free water	4.16 µL
100mM dNTPs (with dTTP)	0.15 µL
MultiScribe™ Reverse Transcriptase, 50 U/µL	1.00 µL
10X Reverse Transcription Buffer	1.50 µL
RNase Inhibitor, 20 U/µL	0.19 µL
Total volume	7.00 µL

* Each 15-µL RT reaction consists of 7 µL master mix, 3 µL of 5X RT primer, and 5 µL RNA sample.

3 Mix gently. Centrifuge to bring the solution to the bottom of the tube.

4 Place the RT mix on ice until you prepare the RNA reaction.

Prepare the RT reaction

5 Thaw the 5X RT primer from the TaqMan® MicroRNA Assays (target and control) on ice.
Thaw the diluted samples (2 ng/µL) on ice.
Before use, vortex the RT primer tubes to mix, then centrifuge briefly.

6 Prepare a 0.2 ml tube for each sample.
Label the lid of the tube.

7 For each 15-µL RT reaction, combine RT mix (from step 2) with miRNA sample and 5X RT primer in the ratio of:

- 7 µL of RT master mix
- 5 µL of miRNA sample (2 ng/µL per reaction)
- 3 µL of 5X RT primer from each assay into the corresponding RT reaction tube.

Note: Before opening the RT primer tubes, thaw the tubes on ice and mix by vortexing, then centrifuge them.

8 Seal the tubes and mix gently.
Centrifuge briefly to bring the solution to the bottom of the tube.

9 Incubate the tube on ice for 5 minutes and keep it on ice until you are ready to load the thermal cycler.

Perform reverse transcription (RT)

10 Note: If applicable, perform the reverse transcription in Standard mode.

Use the following parameter values to program the thermal cycler:

A	B	C
Step	Time	Temperature
Hold	30 minutes	16 °C
Hold	30 minutes	42 °C
Hold	5 minutes	85 °C
Hold	∞	4 °C

11 Set the reaction volume to 15.0 µL.

12 Load the reaction tube or plate into the thermal cycler.

13 Start the RT run.

Note: If you do not immediately continue to PCR amplification after the RT run, store the RT reaction at – 15 to – 25 °C.
