# Supplementary material for: miR-20a is upregulated in serum from domestic feline with PKD1 mutation
Source: PLoS One. 2022 Dec 20;17(12):e0279337. doi: 10.1371/journal.pone.0279337 (PMC9767353; doi:10.1371/journal.pone.0279337)
Supplement: S4 Protocol — (PDF) [file pone.0279337.s005.pdf]

protocols.io

NOV 08, 2022

SHARE

WORKS FOR ME 1

RealTimePCR-Protocol-miRNA-SCALONMC

DOI
dx.doi.org/10.17504/protocols.io.4r3l277z3g1y/v1

Marcela Scalon[1], Christine Souza Martins[1], Gabriel Ginani Ferreira[1], Franciele Schlemmer[1], Ricardo Titze de Almeida[1], Giane Regina Paludo[1]

[1]Universidade de Brasília

Marcela Scalon

Marcela Scalon
Universidade de Brasília

COMMENTS 0

ABSTRACT

This protocol is intended as a guideline to perform the Real Time PCR (qPCR or rtPCR) procedure to detect and quantify miRNAs. This method use TaqMan® MicroRNA Assays and Applied Biosystems real-time PCR instruments. The assays are preformulated primer and probe sets designed to detect and quantify miRNAs, and can detect and quantify small RNA in 1 to 10 ng of total RNA with a dynamic range of greater than six logs. This protocol works on detecting miRNA from samples that were purified from plasma and serum. The amplified products of the RT procedure must be stored – 15 to – 25 °C, if not used immediately for rtPCR. The aim of this protocol is to use a sequence- specific TaqMan® assay to accurately detect miRNAs.

DOI

dx.doi.org/10.17504/protocols.io.4r3l277z3g1y/v1

PROTOCOL CITATION

Marcela Scalon, Christine Souza Martins, Gabriel Ginani Ferreira, Franciele Schlemmer, Ricardo Titze de Almeida, Giane Regina Paludo 2022. RealTimePCR-Protocol-miRNA-SCALONMC. **protocols.io** https://dx.doi.org/10.17504/protocols.io.4r3l277z3g1y/v1

KEYWORDS

plasma miRNA, serum miRNA, rtPCR, probes

LICENSE

This is an open access protocol distributed under the terms of the Creative Commons Attribution License, which permits unrestricted use, distribution, and reproduction in any medium, provided the original author and source are credited

CREATED

Nov 07, 2022

LAST MODIFIED

Nov 08, 2022

PROTOCOL INTEGER ID

72419

GUIDELINES

Target amplification using cDNA as the template is the second step in the two-step RT-PCR. In this step, the DNA polymerase amplifies target cDNA using sequence-specific primers and cleaves the TaqMan® probe to generate a fluorescent signal that is measured by the real-time PCR system.

Keep the TaqMan® assays in the freezer, away from light, until you are ready to use them. Excessive exposure to light may affect the fluorescent probes.

The rtPCR step must be performed on a real-time PCR system.

When preparing samples for PCR amplification:

- Use a positive-displacement pipette or aerosol-resistant pipette tips.
- Follow proper pipette-dispensing techniques to prevent aerosols.
- Wear clean gloves and a clean lab coat (not previously worn while handling amplified PCR products or used during sample preparation).
- Change gloves whenever you suspect that they are contaminated.
- Maintain separate areas and dedicated equipment and supplies for:
    - Sample preparation
    - PCR setup
    - PCR amplification
    - Analysis of PCR products
- Never bring amplified PCR products into the PCR setup area.
- Open and close all sample tubes carefully. Centrifuge tubes before opening. Try not to splash or spray PCR samples.
- Keep reactions and components capped as much as possible.
- Clean lab benches and equipment periodically with 10% bleach solution.

MATERIALS TEXT

Kits and Reagents:

- TaqMan® Fast Advanced Master Mix
- TaqMan® MicroRNA Assays:
  - Tube containing a mix of:
    - Small RNA-specific forward PCR primer
    - Specific reverse PCR primer
    - Small RNA-specific TaqMan® MGB probe
- MicroAmp® Fast Optical 96-Well Reaction Plate
- MicroAmp® Optical Adhesive Film, 100 films

Required laboratory materials and equipment:

- Centrifuge with plate adapter
- Disposable gloves
- Microcentrifuge tubes, 1.5-mL
- Microcentrifuge
- Applied Biosystems Real-Time PCR System
- Pipettors (positive-displacement or air-displacement) and tips:
  - 1- to 20-µL range, 20- to 200-µL range, 100- to 1000-µL range
- Polypropylene tubes
- RNase-free, sterile-filtered water
- Vortexer

SAFETY WARNINGS

Minimize contact with chemicals. Wear appropriate personal protective equipment when handling chemicals (for example, safety glasses, gloves, or protective clothing).

Minimize the inhalation of chemicals. Do not leave chemical containers open. Use only with adequate ventilation (for example, fume hood).

Comply with all local, state/provincial, or national laws and regulations related to chemical storage, handling, and disposal.

BEFORE STARTING

Thoroughly mix the TaqMan® Fast Advanced Master Mix by swirling the bottle.

Thaw frozen TaqMan® assays by placing them on ice. When thawed, resuspend the assays by vortexing, then briefly centrifuge.

Thaw frozen samples by placing them on ice. When thawed, resuspend the samples by briefly vortexing, then briefly centrifuge.

Prepare the real-time PCR (qPCR or rtPCR) reaction mix

1 Determine the total number of PCR reactions to perform. On each reaction plate include:

- A miRNA assay for each cDNA sample
- Control assays
- No template controls (NTCs) for each assay on the plate

Notes:
- It is possible to run multiple assays on one reaction plate. Include controls for each assay.
- Perform 3 replicates of each reaction

2 Per the table below, calculate the total volume required for each reaction component.

Note: Always include extra volume to compensate for the volume loss that occurs during pipetting.

A	B
Component	Volume (µL) for 1 reaction
TaqMan® Fast Advanced Master Mix	10.00 µL
Primer TaqMan® target or control (20X)	1.00 µL
Nuclease-free Water	7.00 µL
Total volume per reaction	18.00 µL

3 Label the microtubes (0.6-mL or 1.5-mL).

4 Add all the components at the calculated quantities.

5 Mix gently, then centrifuge to bring the solution to the bottom of the tube and eliminate air bubbles.

Prepare de PCR reaction plate

6 Transfer 18.00 µL of each PCR reaction mix to each well of an optical reaction plate (MicroAmp® Fast Optical 96-Well Reaction Plate).

7 Add the cDNA or Nuclease-free Water, to the respective well of the plate, as follows:

A	B
Component	Volume (µL) for 1 reaction
cDNA or Nuclease-free Water (for NTC)	2.00 µL
Total volume per well	20.00 µL

8 Cover the reaction plate with an optical adhesive film (MicroAmp® Optical Adhesive Film).

9 Centrifuge the plate briefly to spin down the contents and eliminate air bubbles.

Run the rtPCR reaction plate

10 In the system software, open the plate document or experiment that corresponds to the reaction plate.

11 Load the reaction plate into the real-time PCR system.

12 Set up a plate document or experiment with the following thermal-cycling conditions:

A	B	C

A	B	C
STEP	Temp. (°C)	Time (mm:ss)
Hold	50°C	02:00
Hold	95°C	00:20
PCR (40 cycle	95°C	00:03
	60°C	00:30

13 Start the run.

Analyze the data

14 Data analysis varies depending on the real-time PCR system used.
The general process for analyzing gene expression quantitation data involves:

- Viewing the amplification plots for the entire reaction plate.
- Setting the baseline and threshold values to determine the threshold cycles (CT) for the amplification curves.
- Using the relative standard curve method or the comparative CT method to analyze the data.
