# Supplementary material for: miR-20a is upregulated in serum from domestic feline with PKD1 mutation
Source: PLoS One. 2022 Dec 20;17(12):e0279337. doi: 10.1371/journal.pone.0279337 (PMC9767353; doi:10.1371/journal.pone.0279337)
Supplement: S1 Table — Where Hematocrit (HCT), Red Blood Cell (RBC), Hemoglobin (HGB), Mean corpuscular volume (MCV), Mean corpuscular hemoglobin concentration (MCHC), White blood cell (WBC), Blood Urea Nitrogen (BUN), Phosphorus (P), Potassium (K), Symmetric dimethilarginine (SDMA), Urine Protein/Creatinine Ratio (UPCR), Gamma-Glutamyl Transpeptidase (GGT). (PDF) [file pone.0279337.s006.pdf]

Supporting information Table 1

S1 Table - Mean, standart deviantion (SD) and standart error of the mean (SE) from clincal parameters (blood count, biochemical and urinalysis) of control group.

Hematological parameters	Cats (n)	Mean	SD	SE	Reference values
HCT (%)	10	45.2	3.8	1.2	24-45 †
RBC (x10^6/µL)	10	10.5	1.0	0.3	5-10 †
HGB (g/dL)	10	14.5	1.3	0.8	8-15 †
MCV (fl)	10	43.0	3.4	1.1	39-55 †
MCHC (%)	10	31.9	0.7	0.2	30-36 †
WBC (µL)	10	8900	2600	800	5500-19500 †
Neutrophils	10	4755	1295	409.6	2500-12500 †
Lymphocytes	10	3090	1778	562.2	1500-7000 †
Monocytes	10	137.6	65.7	21.9	0-850 †
Eosinophils	10	882	764	241.6	0-1500 †
Basophils	10	39.3	54.7	17.3	Raros †
Plasma protein (g/dL)	10	7.7	0.4	0.12	6-8 †
Platelet count	9	306667	172076	57359	195000-624000 †

Biochemical parameters	Total	Mean	SD	SE	Reference values
BUN (mg/dL)	10	55.7	6.4	2.0	42.8-64.2 ‡
Creatinine (mg/dL)	10	1.2	0.2	0.1	0.8-1.8 ‡
Total serum protein (g/dL)	10	7.4	0.3	0.1	5.4-7.8 ‡
Albumin (g/dL)	10	4.2	0.4	0.1	2.1-3.3 ‡
Globulin (g/dL)	10				
P (mg/dL)	10	5.3	0.6	0.2	4.5-8.1 §
K	10	4.4	0.5	0.2	4.0-4.5 §
SDMA	10	10.5	1.5	0.5	0-14 #

Urinary parameters	Total	Mean	SD	SE	Reference values
Density	10	1049	7.7	2.5	1035-1060 $
pH	10	6.1	0.9	0.3	5.5-7.5 $
Protein (mg/dL)	10	32.3	8.2	2.6	-
Creatinine (mg/dL)	10	291.8	99.6	31.5	-
UPCR	10	0.1	0.03	0.01	<0.5 $
GGT	10	58.2	29.5	9.3	19-103 ◊

Where Hematocrit (HCT), Red Blood Cell (RBC), Hemoglobin (HGB), Mean corpuscular volume (MCV), Mean corpuscular hemoglobin concentration (MCHC), White blood cell (WBC), Blood Urea Nitrogen (BUN), Phosphorus (P), Potassium (K), Symmetric dimethilarginine (SDMA), Urine Protein/Creatinine Ratio (UPCR), Gamma-Glutamyl Transpeptidase (GGT).

Reference values: † (Jain 1993), ‡ (Kaneko, Harvey, e Bruss 2008), § (Meyer e Harvey 2004), $ (Stockham e Scott 2013), ◊ (UECHI et al. 1998), # (Brown 2016).
