# Supplementary material for: miR-20a is upregulated in serum from domestic feline with PKD1 mutation
Source: PLoS One. 2022 Dec 20;17(12):e0279337. doi: 10.1371/journal.pone.0279337 (PMC9767353; doi:10.1371/journal.pone.0279337)
Supplement: S2 Table — Where Hematocrit (HCT), Red Blood Cell (RBC), Hemoglobin (HGB), Mean corpuscular volume (MCV), Mean corpuscular hemoglobin concentration (MCHC), White blood cell (WBC), Blood Urea Nitrogen (BUN), Phosphorus (P), Potassium (K), Symmetric dimethilarginine (SDMA), Urine Protein/Creatinine Ratio (UPCR), Gamma-Glutamyl Transpeptidase (GGT). (PDF) [file pone.0279337.s007.pdf]

Supporting information Table 2

S2 Table – Mean, standart deviantion (SD) and standart error of the mean (SE) from clincal parameters (blood count, biochemical and urinalysis) of PKD group.

Hematological parameters	Cats (n)	Mean	SD	SE	Reference values
HCT (%)	9	37.8	8.8	2.9	24-45 †
RBC (x10^6/µL)	9	8.9	2.7	0.9	5-10 †
HGB (g/dL)	9	12.1	3.7	1.2	8-15 †
MCV (fl)	9	45.6	7.0	2.3	39-55 †
MCHC (%)	8	32.3	1.0	0.4	30-36 †
WBC (µL)	9	8106	3226	1075	5500-19500 †
Neutrophils	9	5285	3516	1172	2500-12500 †
Lymphocytes	9	1855	1235	411.8	1500-7000 †
Monocytes	9	242.6	211.6	70.5	0-850 †
Eosinophils	9	654.1	636	212	0-1500 †
Basophils	8	27.63	38.31	13.54	Raros †
Plasma protein (g/dL)	9	7.6	0.4	0.1	6-8 †
Platelet count	9	403778	150493	50164	195000-624000 †

Biochemical parameters	Total	Mean	SD	SE	Reference values
BUN (mg/dL)	9	64.2	29.7	9.9	42.8-64.2 ‡
Creatinine (mg/dL)	9	1.4	0.2	0.08	0.8-1.8 ‡
Total serum protein (g/dL)	9	7.4	0.7	0.2	5.4-7.8 ‡
Albumin (g/dL)	9	3.6	0.6	0.2	2.1-3.3 ‡
Globulin (g/dL)	8	3.5	0.4	0.1	
P (mg/dL)	9	5.4	1.3	0.4	4.5-8.1 §
K	9	3.8	1.4	0.5	4.0-4.5 §
SDMA	8	10.2	1.4	0.5	0-14 #

Urinary parameters	Total	Mean	SD	SE	Reference values
Density	7	1035	0.01	0.007	1035-1060 $
pH	7	5.9	0.7	0.3	5.5-7.5 $
Protein (mg/dL)	7	33.8	21.4	8.1	-
Creatinine (mg/dL)	7	248.1	149.5	56.5	-
UPCR	7	0.2	0.2	0.07	<0.5 $
GGT	6	69.2	48.6	21.7	19-103 ◊

Where Hematocrit (HCT), Red Blood Cell (RBC), Hemoglobin (HGB), Mean corpuscular volume (MCV), Mean corpuscular hemoglobin concentration (MCHC), White blood cell (WBC), Blood Urea Nitrogen (BUN), Phosphorus (P), Potassium (K), Symmetric dimethilarginine (SDMA), Urine Protein/Creatinine Ratio (UPCR), Gamma-Glutamyl Transpeptidase (GGT).

Reference values: † (Jain 1993), ‡ (Kaneko, Harvey, e Bruss 2008), § (Meyer e Harvey 2004), $ (Stockham e Scott 2013), ◊ (UECHI et al. 1998), # (Brown 2016).
