# Supplementary material for: miR-20a is upregulated in serum from domestic feline with PKD1 mutation
Source: PLoS One. 2022 Dec 20;17(12):e0279337. doi: 10.1371/journal.pone.0279337 (PMC9767353; doi:10.1371/journal.pone.0279337)
Supplement: S3 Table — Hematocrit (HCT), Red Blood Cell (RBC), Hemoglobin (HGB), Mean corpuscular volume (MCV), Mean corpuscular hemoglobin concentration (MCHC), White blood cell (WBC), Blood Urea Nitrogen (BUN), Phosphorus (P), Potassium (K), Symmetric dimethilarginine (SDMA), Urine Protein/Creatinine Ratio (UPCR), Gamma-Glutamyl Transpeptidase (GGT). (PDF) [file pone.0279337.s008.pdf]

Supporting information Table 3

S3 Table – Clinical parameters (blood count, biochemical and urinalysis) of individual samples.

Sample	HCT (%)	RBC ($x10^6/\mu L$)	HGB (g/dL)	MCV (fl)	MCHC (%)	Platelet count
Control 1	38	9.67	11.8	39	31	752000
Control 2	50	11.71	15.5	43	31	200000
Control 3	44	8.76	14.8	50	33	
Control 4	47	11.29	15.1	42	32	249000
Control 5	46	10.34	14.8	44	32	292000
Control 6	46	11.46	14.5	40	31	239000
Control 7	49	11.98	15.9	41	32	204000
Control 8	48	10.67	15.6	45	32	324000
Control 9	44	9.64	14.3	46	32	280000
Control 10	40	10.02	13.1	40	33	220000
PKD 1	29	6,59	9,4	44	32	580000
PKD 2	38	8,91	12,5	43	33	212000
PKD 3	46	11,9	14,7	39	33	280000
PKD 4	42	9,68	13,9	43	33	334000
PKD 5	23	3,97	4,1	58	18	672000
PKD 6	53	11,42	16,8	46	32	414000
PKD 7	38	10,73	14,4	46	30	405000
PKD 8	35	6,33	11,3	55	32	272000
PKD 9	36	10,12	12	36	33	465000

Sample	WBC (µL)	Neutrophils	Lymphocytes	Monocytes	Eosinophils	Basophils
Control 1	11.1	4440	3885	0	2775	0
Control 2	9.6	5280	3648	96	480	96
Control 3	5.5	3960	880	275	330	55
Control 4	11.9	4046	6545	119	1190	0
Control 5	13.1	7336	4192	131	1441	0
Control 6	7.1	4970	1420	142	426	142
Control 7	6.5	2470	3640	65	325	0
Control 8	6.9	5520	621	138	621	0
Control 9	7.2	4032	2664	72	432	0
Control 10	10.0	5500	3400	200	800	100
PKD 1	14400	13248	864	288	0	0
PKD 2	5200	2496	2444	104	156	0
PKD 3	6700	1675	4151	67	737	67
PKD 4	7300	5110	1241	146	730	73
PKD 5	4200	2940	672	84	462	0
PKD 6	6300	4473	1575	189	63	0
PKD 7	8100	3645	1620	648	2106	81
PKD 8	11750	7050	3408	117	823	352
PKD 9	9000	6930	720	540	810	0

Sample	Plasma protein	BUN	Creatinine	Serum protein	Albumin	Globulin
Control 1	7.4	46	1.1	7.4	3.4	4
Control 2	7.2	52	1.6	7.2	4.3	2.9
Control 3	8.2	47	1.2	7.7	4.6	3.1
Control 4	8.4	63	1.1	8.1	4.6	3.5
Control 5	7.6	59	1.2	7.3	4.2	3.1
Control 6	7.4	55	1.4	7.1	4.6	2.5
Control 7	7.8	62	1.5	7.2	4.4	2.8
Control 8	8.0	53	1.2	7.8	4.0	3.8
Control 9	7.4	64	1.0	7.4	4.1	3.3
Control 10	7.6	56	1.0	7.2	3.9	3.3
PKD 1	7,4	55	1,1	7,2	3,6	3,6
PKD 2	7,4	73	1,4	7,4	3,8	3,6
PKD 3	7,2	84	1,9	7	4	3
PKD 4	7,6	38	1,4	8,6	2,4	6,2
PKD 5	7,2	131	4,1	6	3,3	2,7
PKD 6	8,4	66	1,5	7,7	4,1	3,6
PKD 7	8	50	1,5	7,1	3,2	3,9
PKD 8	7,4	36	1,3	7,3	3,5	3,8
PKD 9	8	45	1,4	7,9	4,4	3,5

Sample	P	K	SDMA
Control 1	6.0	5.16	14
Control 2	4.5	4.7	12
Control 3	6.0	4.43	10
Control 4	5.2	3.91	10
Control 5	5.9	5.03	9
Control 6	5.5	4.16	11
Control 7	4.3	3.87	10
Control 8	5.4	4.8	10
Control 9	4.8	3.5	9
Control 10	6.0	4.66	10
PKD 1	8	6,66	10
PKD 2	4,8	3,86	11
PKD 3	4,8	3,48	10
PKD 4	4,6	3,2	9
PKD 5	6,4	2,37	29
PKD 6	5,9	1,77	10
PKD 7	5,2	3,79	12
PKD 8	3,6	4,33	8
PKD 9	5	4,53	12

Urinary parameters<br>Sample	Density	pH	Protein	Creatinine	UPCR	GGT
Control 1	1039	5.0	17.9	210.58	0.08	92
Control 2	1057	7.5	42	513.9	0.08	54
Control 3	1044	6.0	36.1	386.32	0.09	99
Control 4	1054	6.0	36.9	271.94	0.15	45
Control 5	1060	5.0	31.9	253.02	0.12	105
Control 6	1038	7.0	18.8	211.94	0.08	34
Control 7	1057	6.0	35.2	335.66	0.10	23
Control 8	1049	6.0	37.8	259.02	0.14	46
Control 9	1045	7.5	28.3	177.41	0.16	33
Control 10	1047	5.5	38.4	297.88	0.13	51
PKD 1	1,06	6	30,2	330,58	0,09	50
PKD 2	1,011	5	10,1	16,36	0,62	2
PKD 3	1,046	6	39,2	335	0,12	
PKD 4	1,046	6	31,2	435,58	0,07	128
PKD 5	1,009	5	25,8	76,9	0,33	
PKD 6						
PKD 7	1,037	6.5	22,3	253,65	0,09	64
PKD 8	1,035	7	77,9	288,4	0,27	102
PKD 9						

Hematocrit (HCT), Red Blood Cell (RBC), Hemoglobin (HGB), Mean corpuscular volume (MCV), Mean corpuscular hemoglobin concentration (MCHC), White blood cell (WBC), Blood Urea Nitrogen (BUN), Phosphorus (P), Potassium (K), Symmetric dimethilarginine (SDMA), Urine Protein/Creatinine Ratio (UPCR), Gamma-Glutamyl Transpeptidase (GGT).
