# Supplementary material for: miR-20a is upregulated in serum from domestic feline with PKD1 mutation
Source: PLoS One. 2022 Dec 20;17(12):e0279337. doi: 10.1371/journal.pone.0279337 (PMC9767353; doi:10.1371/journal.pone.0279337)
Supplement: Attachment — Submitted filename: Response to Reviewers.docx [file pone.0279337.s009.docx]

Brasilia, November, 14^th^ 2022

Fabio Sallustio

Academic Editor and Reviewer #1, PLOS ONE

Dear Dr Fabio Sallustio,

The manuscript PONE-D-22-16135, entitled “miR-20a is upregulated in serum from domestic feline with PKD1 mutation” was reviewed. We carefully rechecked and made the appropriate changes in accordance with all suggestions to give further consideration. In addition, we included more details as asked, in the Methods.

The changes made are saved with track changes in Microsoft Word document.

The responses to the comments have been prepared and described below:

**Answers to Reviewer and more details**

1. Comments: “the info about what primer/assays have been used for the miRNA detection is missing and it is mandatory.”
Answer: In **Materials and methods**, Table 1 was added with the complete information about the primer/assays used in the study.

2. Comments: “please include graph of real-time miRNA expression results in the paper and not as supplemental file.”
Answer: In **Materials and methods**, Fig 1 and Fig 2 were added.

3. Comments: “In the graph of miRNA expression results how the fold change has been calculated? Respect to what? It is not clear.”
Answer: In **Materials and methods**, the complete information about how the fold change was calculated is added in lines 155 – 169 on manuscript with track changes document.

4. Comments: “If applicable, we recommend that you deposit your laboratory protocols in protocols.io to enhance the reproducibility of your results.”

Answer: All protocols were deposited to Protocols.io, and are provided as supplemental files.

5. Comments: “In the ethics statement in the Methods and online submission information, please specify whether cat owners' consent was obtained and (2) what type you obtained (for instance, written or verbal, and if verbal, how it was documented and witnessed). If the need for consent was waived by the ethics committee, please include this information”

Answer: In **Materials and methods**, the information about the consent form was added, and a copy with the text (in Portuguese and English) is provided as a supplemental file.

6. Comments: “The 5 miRs selected in this study based on a previous study of miRs of kidney specimens from ADPKD cats, detailed description of these 5 miRs association with ADPKD were absent and needs to be added.”
Answer: In lines 70 – 90 of **Introduction**, the detailed descriptions of the 5 miRs and its association with ADPK were added.

7. Comments: “The role of miR-20a on cystic formation in ADPKD cats was inferred by the author. However, the relation between serum levels of miR-20a and renal cysts formation or different disease stages were absent.”
Answer: In **Results and discussion**, the information about miR-20a serum levels and renal cysts formation was added in lines 230 – 235 on manuscript with track changes document.

8. Comments: “The bioinformatics of miR-20a and its target genes have not been fully discussed.”
Answer: In **Results and discussion**, the bioinformatics of miR-20a and its target genes was added in lines 244 – 249 on manuscript with track changes document.

9. Comments: “As none coding RNAs is species and genus specific, the deduction (line 37-38) “better understanding of relative expression profiles will further enhance the development of novel diagnostic and prognostic biomarkers as well as prompting patient management” is inappropriate.”
Answer: In **Abstract**, the sentence was removed.

10. Comments: “While revising your submission, please upload your figure files to the Preflight Analysis and Conversion Engine (PACE) digital diagnostic tool, <https://pacev2.apexcovantage.com/>.”
Answer: All figures were uploaded to PACE.

We thank you and the reviewer for your thoughtful suggestions and insights, which have enriched the manuscript and produced a more balanced and better account of the research. We hope that the revised manuscript is now suitable for publication in your journal.

I look forward to your reply.

Sincerely,

Giane Regina Paludo,

College of Agronomy and Veterinary Medicine,

University of Brasilia, ICC Sul, Campus Universitário Darcy Ribeiro,

Brasília, DF 70910-970, Brazil.

[giane@unb.br](mailto:giane@unb.br)
